# Intra-subject consistency of spontaneous eye blink rate in young women across the menstrual cycle

**DOI:** 10.1038/s41598-020-72749-2

**Published:** 2020-09-24

**Authors:** Esmeralda Hidalgo-Lopez, Georg Zimmermann, Belinda Pletzer

**Affiliations:** 1 Department of Psychology and Centre for Cognitive Neuroscience, University of Salzburg, Hellbrunnerstr. 34, 5020 Salzburg, Austria; 2 Team Biostatistics and Big Medical Data, IDA Lab Salzburg, Paracelsus Medical University Salzburg, Strubergasse 21, 5020 Salzburg, Austria

**Keywords:** Psychology, Human behaviour, Cognitive neuroscience, Oculomotor system

## Abstract

The spontaneous eye blink rate (EBR) has been linked to different cognitive processes and neurobiological factors. It has also been proposed as a putative index for striatal dopaminergic function. While estradiol is well-known to increase dopamine levels through multiple mechanisms, no study up to date has investigated whether the EBR changes across the menstrual cycle. This question is imperative however, as women have sometimes been excluded from studies using the EBR due to potential effects of their hormonal profile. Fifty-four women were tested for spontaneous EBR at rest in three different phases of their menstrual cycle: during menses (low progesterone and estradiol), in the pre-ovulatory phase (when estradiol levels peak and progesterone is still low), and during the luteal phase (high progesterone and estradiol). No significant differences were observed across the menstrual cycle and Bayes factors show strong support for the null hypothesis. Instead, we observed high intra-individual consistency of the EBR in our female sample. Accordingly, we strongly encourage including female participants in EBR studies, regardless of their cycle phase.

## Introduction

For more than 70 years^1^, the spontaneous eye blink rate (EBR) has been used as a physiological measure related to diverse neurocognitive and biological factors^2^. These factors range from individual genetic make-up^3^ and neuropsychiatric disorders^4^, to psychological personality traits^5,6^, attentional regulation^7^, learning processes^8,9^, cognitive flexibility^3,10^ and other executive functions^11^. Some of these effects have been suggested to reflect dopaminergic functioning^12–14^. Given its non-invasive nature, the EBR has been used as a proxy for striatal dopamine (DA) levels as an alternative to direct measurements like positron emission tomography (PET) and single-photon emission computer tomography (SPECT)^15^.

Converging evidence from animal and human studies show a positive correlation of the EBR to DA levels in the striatum. For instance, the EBR correlates positively with DA levels in the caudate nucleus of non-human primates^16^. A number of studies have shown that DA agonists and antagonists increase and decrease EBRs, respectively, both in animals^17–20^, and healthy humans^21–23^. Likewise, studies in human patients with abnormal dopaminergic function show a reduced EBR for Parkinson’s disease^4,12,24^ and increased EBR for schizophrenia^25,26^ and Tourette syndrome^27^. Additionally, reduced EBR has been proposed as putative index for reduced D2/3-receptor availability, in relation to chronic drug consumption^28^, increased alcohol/nicotine use, and gambling problem severity^29^ However, little is known about the neural circuitry underlying inter-individual differences in the EBR. Kaminer et al.^19^ suggested a model for humans and rodents, in which the trigeminal complex is altered by striatal DA levels, changing the EBR. This relation to the dopaminergic system is not without inconsistencies and some studies reported evidence to the contrary^30,31^. A causal relationship has recently been established between the right angular gyrus and the EBR^32^. The gray matter volume in the right angular gyrus was positively correlated to the EBR, and a disruption of activity in this area by transcranial magnetic stimulation decreased the EBR.

Independently of the neural basis of the EBR, several factors have been consistently reported to modulate it. Among others, the EBR has been observed to change following a circadian^33^ and seasonal rhythm^34^, during different cognitive states^35^ and with age, although not in a consistent pattern for the latter. An increase from childhood to maturity appears to be steady^11,36–38^, but results are inconsistent regarding the age-related decline. Specifically, age seems to interact with sex, another factor reported to modulate the EBR. Around menopause, women experience a significant drop in EBR, whereas in men, the EBR decreases steadily^39^. Although sex differences are not consistently reported^6,11,40–42^, studies demonstrating a main effect of sex, usually report higher EBR in women than in men^3,8,9,43–45^. Some of these effects have been attributed to endogenous variations in ovarian hormone levels of women throughout their life span^39^. Furthermore, endogenous fluctuations in ovarian hormones across the menstrual cycle have been assumed to play a role in EBR modulation^46^. However, the relationship of the EBR to such endogenous hormone fluctuations has never been explicitly researched.

Indeed, both estradiol and progesterone have neuroactive effects and are known to modulate dopaminergic functioning^47^. Specifically, estradiol increases the synthesis, release, reuptake and turnover of DA in the prefrontal cortex and the striatum and modifies basal firing rates of dopaminergic neurons^48–52^. Research on progesterone’s effects on the other hand, is inconsistent and scarce^53^. For instance, in the striatum, progesterone has been shown either to potentiate and enhance estrogenic actions, or to oppose them (thus inhibiting DA release); depending on the mode of administration, concentration and prior administration of oestrogens^54,55^. While these effects are well-known, especially in animal research, up to date no study has investigated the EBR across the menstrual cycle. In naturally cycling women, ovarian hormone levels fluctuate over approximately 28–29 days and three different hormonal milieus can be distinguished. At the beginning, during menses, circulating levels of estradiol and progesterone are still low. Then estradiol starts to rise and peaks right before ovulation, while progesterone is still low. After ovulation, the luteal phase is characterized by increasing levels of progesterone and medium estradiol levels^56^. However, some of the studies including the EBR as putative index for DA system only include males^5,29,57^, and others explicitly state that female sex hormones could affect their measurements^58^. This sex bias towards male subjects is even more pronounced in animal research (^16,59^ in monkeys^19^, in rodents). Human EBR studies that include women do not control for menstrual cycle^9,45^, or do so very loosely^34^.

Given this conjuncture in which no research has been carried out to date, but still scientists face the decision on how to deal with female subjects, it is of the uttermost importance to determine if and how the EBR changes across the menstrual cycle. Based on previous literature supporting the enhancing effect of estradiol on striatal DA function, and the positive relationship between the latter and the EBR, we expect the EBR to increase during the pre-ovulatory phase, when estradiol is high and progesterone low.

## Materials and methods

### Participants

Eighty-three young healthy right-handed women were recruited from the University of Salzburg and through social media, as well as from the samples of previous studies. Twenty-nine participants were excluded prior to analyses because of inconsistencies between hormone values and cycle phase as calculated based on self-reports (see “Hormone analysis” section). Therefore, analyses were performed in 54 women with an age range between 18 and 35 years (*M*_age_ = 23.95, *SD* = 3.68). We specifically used this range in order to maintain the sample as homogeneous as possible, given that sex hormones levels vary after 35 years old^60^, and an EBR decline has been related to perimenopause in women^39^. All of them had a regular menstrual cycle (*M*_cycle length_ = 28.63 days*, SD* = 2.24) and had not used hormonal contraceptives within the previous 6 months. Regular menstrual cycle was defined as ranging between 21 and 35 days and a variability of cycle length between individual cycles of less than 7 days^56^. Other exclusion criteria were neurological, psychiatric or endocrine disorders, and being under medication treatment.

### Ethics statement

Experiments were approved by the local ethics committee and were conducted in accordance with the Code of Ethics of the World Medical Association (Declaration of Helsinki), and all participants gave their informed written consent to participate in the study. Upon arrival at the lab, participants were assigned a subject ID (VP001, VP002, etc.), which was used throughout the study.

### Procedure

Three different sessions were scheduled for each participant, time-locked to their menstrual cycle, in order to study each of the hormonal milieus, as described in^61^. Therefore, appointments were scheduled (i) during menses (low progesterone and estradiol), (ii) in the pre-ovulatory phase (when estradiol levels peak and progesterone is still low), and (iii) during the mid-luteal phase (high progesterone and estradiol), order counter-balanced. For cycle phase estimation, cycle-length was estimated based on participants self-reports of the onsets of their last three menstrual periods. Since irrespective of cycle length, the duration of the luteal phase is relatively stable around 14 days^56^, ovulation was estimated to fall 14 days before the onset of next menses. Menses sessions spanned from the first to the seventh day of menstruation (*M*_day_ = 3.78, *SD* = 1.37) depending on the individual cycle length. Pre-ovulatory sessions were scheduled 2–3 days before the expected date of ovulation and confirmed by commercially available urinary ovulation tests (PREGNAFIX) (*M*_day_ = 12.26, *SD* = 2.29). Mid-luteal sessions were scheduled in a window ranging from day 3 post ovulation to 3 days before the onset of the next menstruation (*M*_day_ = 21.67, *SD* = 2.83). Cycle phases were additionally confirmed by salivary hormone levels and participants were excluded if the levels were not as expected for both hormonal values: estradiol not the lowest during menses and progesterone not the highest during luteal phase (see “Hormone analysis” section).

Given that eye blink rate (EBR) is supposed to be stable during daytime, but increases in the evening^33^, every session took place between 8 am and 5.30 pm. The three sessions of each participant were scheduled approximately at the same time of the day, and time was included as a control variable into the analyses to avoid any confounding effect (see “Statistical analyses” section). Before each session, participants filled in a brief questionnaire concerning food, sports, sleeping habits, and possible ongoing stressors. Spontaneous EBR was recorded as follows. Participants were seated 1 m from a white wall with a black cross at their eyes height and asked to fix their gaze in resting conditions. Vertical and horizontal electro-oculograms (EOGs) were recorded with an EEG system (actiCAP, Brain Products GmbH, Germany) at a sampling rate of 500 Hz and impedances kept under 50 kΩ. Active skin electrodes were placed above the right orbita and at the outer canthi from both eyes, referenced against an electrode below the right orbita, and with a grounding electrode placed on the forehead. Each eye blink was defined as an amplitude wave with a voltage change of 100 µV in a time interval of 500 ms^62^. The recording lasted six consecutive minutes and the EBR was defined as the average number of blinks per minute. Signals were amplified using an ActiCHamp Amplifier (Brain Products GmbH, Germany) and the posterior analysis of the recorded blinks was performed online with Brain Vision Analyzer 2.1 (Brain Products GmbH, Munich, Germany). Two independent observers visually scored the number of blinks for the 6 min segment with an inter-rater reliability of 99% (Cronbach's α > 0.99). After excluding two observations in which the participants blinked at an abnormally high rate (more than 3 *SD*), EBR ranged from 1.17 to 49.17. Removal of outliers did not produce any substantial changes in the cycle phase effect.

### Hormonal analysis

In order to assess estradiol and progesterone levels procedure was followed as described in^63^. Two saliva samples, each of 2 ml volume, were collected one before and the second after each session and stored in a freezer at − 20 °C immediately after collection. Prior to analysis solid particles were removed by centrifugation (3000 rpm for 15 min, then 3000 rpm for 10 min). Saliva from the two samples was pooled before the analyses to provide a more stable assessment for the average of the hormone levels. Estradiol and progesterone levels were quantified using ELISA kits from DeMediTec Diagnostics, Kiel, Germany. Sensitivity was 0.6 pg/ml for estradiol and 5.0 pg/ml for progesterone. According to the information provided by the manufacturer intra-assay coefficient of variation (CV) was between 2.4% and 8.3% for estradiol and between 6.0 and 9.6% for progesterone. Inter-assay CV was between 2.8% and 12.0% for estradiol and between 8.6 and 10.1% for progesterone. All samples were run in duplicates and assessment of samples with more than 25% variation between duplicates was repeated. Hormone values were used to exclude participants with a mismatch between the actual and expected hormonal profile. Due to insufficient sample volume, there were two missing values for the hormone levels. Each missing value belonged to two different participants and neither of them corresponded to the luteal phase.

### Statistical analyses

Statistical analyses were performed in R 3.6.2 (https://www.R-project.org/)^64^ using *nlme*^65^ and *BayesFactor* packages with default non-informative priors^66^. The variable *time of the day* ranged from 8.00 am to 5.30 pm, and was converted into a categorical variable by splitting it into four groups, according to the quartiles of the sample (i.e., p_25_ = 11:50 am, p_50_ = 1:00 pm, p_75_ = 2:30 pm). Moreover, for subsequent modeling, metric variables were standardized prior to the respective analyses. Statistical significance was defined as p < 0.05.

At first, to explore the menstrual cycle effects on the EBR, a linear mixed model was fitted to the data, using *EBR* as dependent variable, *cycle phase* and *time of the day* as fixed effects, *age* as a fixed covariate, and *participant number* (PNr) as random effect, respectively. Formally, this corresponds to the model equation

$$ {\text{Y}}_{{{\text{ijkl}}}} = \, \mu \, + {\text{ P}}_{{\text{i}}} + {\text{ C}}_{{\text{j}}} + {\text{ T}}_{{\text{k}}} + \, \beta {\text{A}}_{{\text{i}}} + \, \varepsilon_{{{\text{ijkl}}}} , $$

where Y_ijkl_ is the eye blink rate of the l-th observation, μ is the population mean, P_i_ is the random effect of participant i, C_j_ is the fixed effect of cycle number (j = 1, 2, 3), and T_k_ denotes the fixed effect corresponding to time of the day (k = 1, 2, 3, 4). Finally, the age of participant i is denoted by A_i_, and ε_ijkl_ is the residual (or error) term. Our main interest lies on testing the effect of cycle phase, adjusting for the other variables included in the model. An unspecific covariance structure was chosen, thereby allowing for heteroscedasticity and varying correlations between cycle phases. Analogous models, yet with eye blink rate being replaced by estradiol and progesterone, respectively, were used for assessing changes in hormone levels across the menstrual cycle.

We accounted for multiple testing by using the package *multcomp*^67^ for conducting all-pairwise comparisons between cycle phases. Moreover, since the sample sizes were somewhat limited, the assumptions of linearity and normality were difficult to assess in a reliable way. Therefore, we conducted an additional sensitivity analysis by using the RM function in the *MANOVA.RM*^68^ package in R. The underlying model also allows for including within- and between-subject factors, with the only difference compared to the linear mixed model that age had to be dichotomized by applying a median split. We extracted the ANOVA-type (AT) and the Wald-type (WT) permutation test statistics with the corresponding p-values, because these tests are expected to perform well even under violations of the normality assumption^69^.

In order to further examine the potential impact of cycle phase on EBR, we additionally applied a Bayesian approach, comparing the specific models of interest (i.e., with and without cycle phase). The Bayes factor (BF) quantifies the relative likelihood of the observed data under two competing models. Let H_0_ denote the null hypothesis (i.e., model without cycle phase), and H_1_ the alternative hypothesis (model including the cycle phase), respectively. Then, the BF is defined as follows^70^:

$${BF}_{01}= \frac{\text{likelihood\,of\,data\,given }{H}_{0}}{\text{likelihood\,of\,data\,given }{H}_{1}}$$

To test the random effect of the participant number (PNr) we compared the full model H_1_ to a model without participant number H_0′_, as described in^66^. When using the *BayesFactor* package, the number of iterations for Monte Carlo sampling was set to the default value (i.e., 10,000)^66^.

## Results

Overall, the sample consisted of n = 54 women with a mean age of about 24 years. Further descriptive statistics regarding basic variables, the hormone levels and the eye blink rates are displayed in Table 1.

**Table 1:** Demographic data, hormone levels and eye blink rate during each cycle phase.

Sample (n = 54)	Age (y.o.)	Cycle length (days)	First session	Cycle day of assesment	Estradiol (pg/ml)	Progesterone (pg/ml)	Eye Blink Rate
Menses	23.95 $\pm$ 0.50	28.63 $\pm$ 0.31	22	3.78 $\pm$ 0.19	2.98 $\pm$ 0.15	72.51 $\pm$ 8.93	15.48 $\pm$ 1.56
Pre-ovulatory			20	12.26 $\pm$ 0.32	3.50 $\pm$ 0.19	93.80 $\pm$ 12.12	16.14 $\pm$ 1.81
Luteal			12	21.67 $\pm$ 0.40	3.69 $\pm$ 0.19	283.98 $\pm$ 33.38	16.11 $\pm$ 1.59

Values are presented as mean $\pm$ standard error of the mean (M $\pm$ SEM) for the final sample of n = 54.

### Endocrine results

Estradiol was significantly higher in the pre-ovulatory phase and luteal phase compared to menses (*p* < 0.001; for details see Tables S1, S2 in the supplement), yet did not differ significantly between pre-ovulatory and luteal phase (p = 0.325). Progesterone was significantly higher in the luteal phase compared to menses and pre-ovulatory phases (*p* < 0.001; for details see Tables S3, S4 in the supplement), but did not differ significantly between the pre-ovulatory phase and menses (*p* = 0.130).

### Menstrual cycle changes in spontaneous eye blink rate

**Figure 1:**
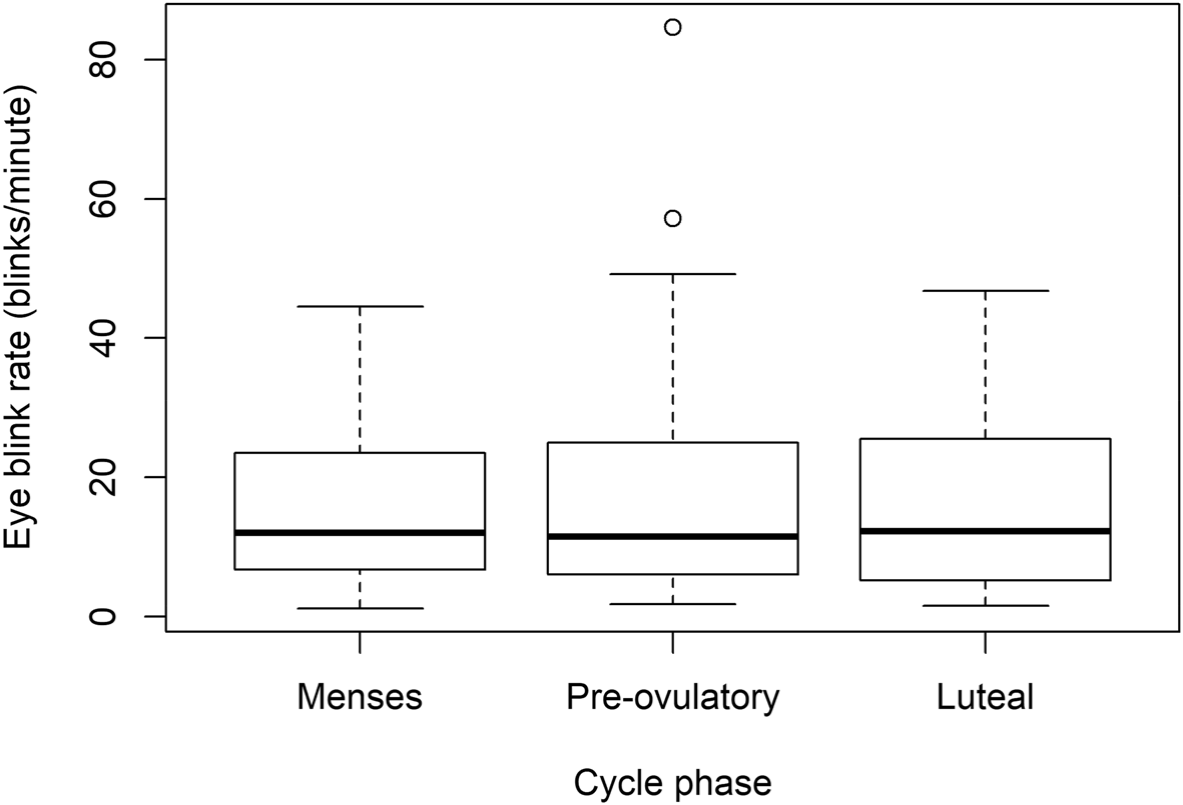
Bloxplot of the eye blink rate along the menstrual cycle (outliers included): Blinks per minute along the three cycle phases did not change significantly (p = 0.421).

No significant differences in EBR were found across the menstrual cycle, nor changes related to the time of the day or age (Fig. 1, Table 2). The standard deviation corresponding to the random subject effect was equal to 0.856, and the residual standard deviation was 0.4904. Multiple pairwise comparisons between cycle phases revealed that the most pronounced, yet non-significant difference between EBRs occurred between menses and pre-ovulatory phases (Table 3). Moreover, the results from the analysis using the *MANOVA.RM* package were overall in line with the findings from the linear mixed model, including a non-significant effect of the cycle phase (WTS, p = 0.620 and ATS, p = 0.621). The trend observed for age in the linear mixed model was not corroborated by the sensitivity analysis.

**Table 2:** Linear mixed model results for the fixed effects, using eye blink rate as outcome variable.

Variable	F value	DF_n_, DF_d_	p value
Time of the day	0.947	3, 101	0.421
Age	4.004	1, 52	0.051
Cycle phase	1.080	2, 101	0.343

*F value* ANOVA test statistic, *DF*_*n*_ numerator degrees of freedom, D*F*_*d*_ denominator degrees of freedom.

**Table 3:** All-pairwise comparisons of eye blink rates between cycle phases.

Comparison	Mean difference (95% CI)	Test statistic	p value
P minus M	0.137 (− 0.082, 0.355)	1.466	0.306
L minus M	0.060 (− 0.129, 0.249)	0.741	0.738
L minus P	− 0.077 (− 0.267, 0.114)	− 0.944	0.611

Mean differences and confidence intervals (CI) refer to differences in (standardized) eye blink rates.

*M* menses, *P* pre-ovulatory, *L* luteal.

p values and confidence intervals have been adjusted for multiple comparisons.

In order to quantify the support of a model without cycle phase relative to a model including it (i.e., the “full model”, see “Statistical analyses” section) we applied a Bayesian approach. The resulting Bayes Factor BF_01_(H_0_/H_1_) = 6.410 ± 3.41% again indicated that cycle phase did not play a significant role with respect to eye blink rate, instead providing substantial evidence^71^ for the model without cycle phase. Finally, in order to corroborate the intra-subject consistency of the EBR (between-session reliability, Cronbach’s α > 0.90), we quantified the support of a model without the participant number relative to a model including it. The resulting BF_0′1_(H_0′_/H_1_) = 9.422 e^−27^ ± 2.46%, indicated very strong evidence in favor of the random effect of the PNr in the EBR^71,72^.

## Discussion

The aim of the present study was to explore the spontaneous eye blink rate (EBR) across the menstrual cycle. Given that the EBR has been implicated in dopamine-dependent cognitive processes^2^ and estradiol has been shown to enhance dopamine levels^48–52^, we hypothesized that EBR would increase during the high estradiol pre-ovulatory cycle phase. Contrary to our expectations, we observed substantial evidence supporting a model without the cycle phase over the model including it^71–73^. We also provided very strong and decisive support^71,72^ for the intra-subject consistency of the EBR in women, and thus, we conclude that the fluctuation of endogenous ovarian hormone levels is not reflected on EBR measurements along the menstrual cycle.

Although changes in the EBR depending on women’s hormonal status have been previously reported, like in women on the contraceptive pill^41^ or postmenopausal women^39^, the subtle variation of endogenous hormones during the reproductive age does not seem to impact the EBR. Accordingly, we did not find any relation of the EBR measures across the cycle phases to estradiol or progesterone levels. Even when hormonal changes across the menstrual cycle have been linked to different dopamine (DA) baseline levels^74,75^, the EBR does not seem sensitive to those changes, probably given its high intra-subject consistency. Moreover, the sensitivity of the EBR as a physiological measure for striatal DA levels is still under debate. Although inter-individual variation in the EBR has been consistently linked to dopaminergic functioning^2^, it remains a highly unspecific, though non-invasive, measure. On one hand, converging evidence from animal and human research attributes the relationship between EBR and DA to the tonic striatal levels and D2 type receptor^44,59,76,77^, mainly expressed in striatum^78^. On the other hand, some PET studies in humans did not find a relation between EBR and DA synthesis capacity^31^ or D2/3-receptor availability^30^. More recently, in a pharmacological study, Chakroun et al.^58^ found no modulation of the EBR by L-dopa (DA precursor) and haloperidol (D2 receptor antagonist).

More importantly, the present results do not support the exclusion of female participants when using the EBR. Women continue to form the smaller proportion of subjects in scientific research, and, while in animal research sex and endocrine status is usually controlled, this does not apply to humans. Paradoxically, studies including human and non-human species, controlled for sex in animals, but not in humans^19^. Despite some inconsistent results^6,11,40–42^, strong evidence points to higher EBR in women compared to men (see review^46^), which could reflect the higher extracellular baseline levels of DA in women^79^. Given these possible sex differences and the impact of sex hormones on the DA system, especially important for DA-dependent neuropsychiatric syndromes^80^, women should be included in future studies and the factor sex should be taken into account. Although in a previous study we used the EBR during menses as a cautionary measure^63^, the present results evidence the stability of the EBR also across the menstrual cycle.

### Conclusions

In summary, EBR appears to be highly stable within healthy young women and a noisy putative measure for striatal dopaminergic functioning, probably reflecting a trait-like stability more than dynamic changes^42,59^. The present study shows that a possible effect derived from the endogenous fluctuation of sex hormones can no longer be used as deterrence against including female participants. Therefore, we strongly encourage researchers to include women, regardless of their cycle phase, in future EBR research.

## Supplementary information

Supplementary Information

## Data Availability

Data and scripts will be openly available at https://webapps.ccns.sbg.ac.at/OpenData/.
