# Supplementary material for: Intra-subject consistency of spontaneous eye blink rate in young women across the menstrual cycle
Source: Sci Rep. 2020 Sep 24;10:15666. doi: 10.1038/s41598-020-72749-2 (PMC7519086; doi:10.1038/s41598-020-72749-2)
Supplement: Supplementary file 1 — Supplementary Information [file 41598_2020_72749_MOESM1_ESM.docx]

**Intra-subject consistency of spontaneous eye blink rate in young women across the menstrual cycle.**

Esmeralda Hidalgo-Lopez^a*^, Georg Zimmermann^b^, Belinda Pletzer^a*^

^a^Department of Psychology and Centre for Cognitive Neuroscience,

University of Salzburg, Hellbrunnerstr. 34, 5020 Salzburg, Austria

^b^ Team Biostatistics and Big Medical Data, IDA Lab Salzburg, Paracelsus Medical University Salzburg, Strubergasse 21, A-5020 Salzburg, Austria

*Corresponding authors:

Esmeralda Hidalgo-Lopez: esmeralda.hidalgolopez@sbg.ac.at (Hidalgo-Lopez, E.)

Hellbrunnerstr. 34, 5020 Salzburg

0043-662-8044-5178

Belinda Pletzer: Belinda.Pletzer@sbg.ac.at (Pletzer, B.)

Hellbrunnerstr. 34, 5020 Salzburg

0043-662-8044-5184

**Supplementary material**

| **Variable** | **F value** | **DF_n_, DF_d_** | **P value** |
| --- | --- | --- | --- |
| Time of the day | 0.06230 | 3, 103 | 0.980 |
| Age | 0.14906 | 1, 52 | 0.701 |
| Cycle phase | 27.82315 | 2, 103 | <0.001 |

**Table S1.** **Linear mixed model results for the fixed effects, using estradiol as outcome variable:** F value = ANOVA test statistic, DF_n_ = numerator degrees of freedom, DF_d_ = denominator degrees of freedom.

| **Comparison** | **Mean difference (95% CI)** | **Test statistic** | **P value** |
| --- | --- | --- | --- |
| P minus M | 0.390 (0.239, 0.542) | 5.989 | <0.001 |
| L minus M | 0.534 (0.315, 0.754) | 5.670 | <0.001 |
| L minus P | 0.144 (-0.093, 0.382) | 1.417 | 0.325 |

**Table S2.** **All-pairwise comparisons of estradiol between cycle phases.** Mean differences and confidence intervals (CI) refer to differences in (standardized) estradiol values. M = menses, P = pre-ovulatory, L = luteal. P values and confidence intervals have been adjusted for multiple comparisons.

| **Variable** | **F value** | **DF_n_, DF_d_** | **P value** |
| --- | --- | --- | --- |
| Time of the day | 1.08047 | 3, 101 | 0.361 |
| Age | 1.10652 | 1, 52 | 0.298 |
| Cycle phase | 23.93789 | 2, 101 | <0.001 |

**Table S3.** **Linear mixed model results for the fixed effects, using progesterone as outcome variable:** F value = ANOVA test statistic, DF_n_ = numerator degrees of freedom, DF_d_ = denominator degrees of freedom.

| **Comparison** | **Mean difference (95% CI)** | **Test statistic** | **P value** |
| --- | --- | --- | --- |
| P minus M | 0.117 (-0.026, 0.259) | 1.865 | 0.130 |
| L minus M | 1.157 (0.760, 1.554) | 6.648 | <0.001 |
| L minus P | 1.040 (0.696, 1.384) | 6.897 | <0.001 |

**Table S4.** **All-pairwise comparisons of progesterone between cycle phases.** Mean differences and confidence intervals (CI) refer to differences in (standardized) progesterone values. M = menses, P = pre-ovulatory, L = luteal. P values and confidence intervals have been adjusted for multiple comparisons.
